# Molecular systematics and distribution review of the endemic cyprinid species, Persian chub, *Acanthobrama persidis *(Coad, 1981) in Southern Iran (Teleostei: Cyprinidae)

**Published:** 2015-12

**Authors:** Azad Teimori, Hamid Reza Esmaeili, Golnaz Sayyadzadeh, Neda Zarei, Ali Gholamhosseini

**Affiliations:** 1 Department of Biology, Faculty of Sciences, Shahid Bahonar University of Kerman, Kerman, Iran.; 2 Ichthyology Research laboratory, Department of Biology, College of Sciences, Shiraz University, Shiraz, Iran; 3 Department of Biology, College of Sciences, Shiraz University, Shiraz, Iran

**Keywords:** mt-DNA, Persian Chub, *Pseudophoxinus*, *Petroleuciscus*, Iranian drainages

## Abstract

The Iranian Persian chub is an endemic species of the family Cyprinidae known only from few localities in drainages of Southern Iran. It was originally described in the genus *Pseudophoxinus* as (*Pseudophoxinus persidis*) and then *Petroleuciscus* (as *Petroleuciscus persidis*). In this study, we examined phylogenetic relationships of the Iranian Persian chub with other relatives in the family Cyprinidae based on the mitochondrial cytochrome b gene to estimate the phylogenetic (and taxonomic) position of the species. Our molecular phylogenies show that new fish sequences from the drainages in southern Iran are clustered with sequences of the genus *Acanthobrama* from GenBank while the sequences from two other genera (*Pseudophoxinus* and *Petroleuciscus*) are in distinct clade. Therefore, we conclude that the populations of Persian Chub in drainages of southern Iran (i.e., Kol, Kor, Maharlu and Persis) belong to the genus *Acanthobrama *and species* Acanthobrama persidis. *The predicted geographic distributions for the species showed a large area of suitable climate for *A. persidis* across south and west of Iran especially in the Kor River basin. Some other parts in the Persis and Tigris are also might have been suitable habitats for this cyprinid species showing possible dispersal route of *Acanthobrama* from Tigris to the Persis, Kor and Kol basins.

## INTRODUCTION

**Figure 1:**
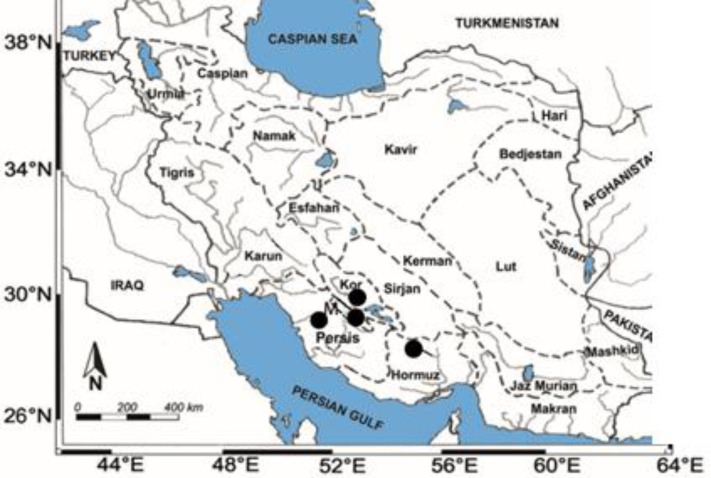
Drainage basins of Iran showing locations of the studied *Acanthobrama persidis *populations. M, Lake Maharlu basin. Sources of maps: Coad (2014) with modification

The confirmed freshwater ichthyofauna of Iran are represented by 202 species in 104 genera, 28 families, 17 orders and 3 classes found in 19 different basins (Fig. 1) [1]. The most diverse order is the Cypriniformes with 120 confirmed species (59.4%) including Cyprinidae with 93 confirmed species (46.0%), Nemacheilidae with 22 species (10.9%) and Cobitidae with 5 species (2.5%) [1]. However there have been controversial debates on the taxonomic status of some endemic cyprinid fishes e.g., *Petroleuciscus persidis* (Coad, 1981) [2].

*Petroleuciscus persidis* was originally described as *Pseudophoxinus persidis* by Coad (1981) [2]. Trewavas (1972) [3] discussed the generic nomenclature of *Pseudophoxinus* Bleeker, 1859 in which this species was originally described. It was treated as *Pseudophoxinus persidis* [4] and considered as more closely related to the genus *Leuciscus* [5]. In addition, Bogutskaya (1996) [5] placed this species in her *Leuciscus borysthenicus* (Kessler, 1859) group which also includes *L. ulanus* and deserves subgeneric rank, but later erected a new genus *Petroleuciscus* which is distinguished by the small size of adults, the reduced number of vertebrae (modally 34-38 in total, rarely 39 or 40), few sensory cephalic pores (7-10 in the supraorbital canal, 12-19 in the infraorbital canal and 12-17 in the preoperculo-mandibular canal), a relatively small supraethmoid-mesethmoid block, narrow infraorbitals, and a deep neurocranium with a normally developed interorbital septum [6-7]. This species was considered as *Petroleuciscus persidis* [1, 8]. 

The cyprinid genus *Petroleuciscus *Bogutskaya, 2002 [6, 9] has three species in Iran, namely *Petroleuciscus esfahani* Coad and Bogutskaya, 2010 [9] from central Iran in the Zayandeh River basin, *P. ulanus *(Günther, 1899) [10] endemic to the Lake Orumiyeh (Urmia) basin in northwestern Iran and *P. persidis *(Coad, 1981) [2] of the endorheic Kor River and the exorheic Kol (Hormuz) basin in southern Iran [11]. *Petroleuciscus gaderanus *(Günther, 1899) [10] is an endemic taxon of the Lake Orumiyeh basin tentatively considered a synonym of *P. ulanus *[9]. 

Perea et al. (2010) [12] using mitochondrial and nuclear DNA concluded that *Petroleuciscus* is not monophyletic and *P. persidis* was included with *Abramis*, *Acanthobrama*, *Acanthalburnus*, *Ballerus*, *Blicca*, *Mirogrex* and *Vimba*. 

The type locality of *Pseudophoxinus persidis* is the "upper Shur River drainage at "Koorsiah" village, near Darab on Darab-Fasa road, 28°45.5'N, 54°24'E, Fars" [2] where no specimens were collected during the past 15 years. The holotype is a 54.7 mm standard length male held at the Canadian Museum of Nature, Ottawa under CMNFI 1979-0154A. Paratypes comprise 95 fish, 34.7-58.8 mm standard length, from the same locality as the holotype under CMNFI 1979-0154B, and 5 fish, 74.7-92.4 mm standard length, under CMNFI 1979-0499 from an "irrigation ditch at village 32 km west of Kor River Bridge on road to Dariush (Dorodzan) Dam, 30°04.5'N, 52°36'E, Fars" [2]. 

**Figure 2:**
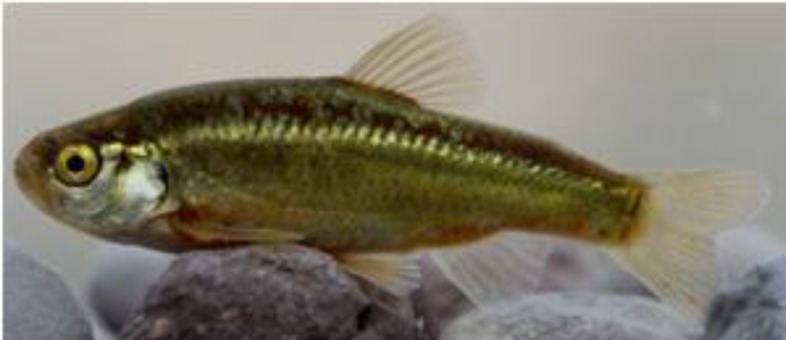
Live specimen of *Acanthobrama persidis *from upper reaches of Kor River basin

The morphology of this species is showed in Figure 2. It is characterized by having a pharyngeal tooth count of 1,5-4,1, modally 7 branched dorsal fin rays, anal fin branched rays 7-9, pelvic fin rays 7-8, pored lateral line scales 35-43 in a complete lateral line, short gill rakers numbering 10-14 on the whole first arch, total vertebrae 34-37, a light coloured peritoneum but with numerous melanophores, and flanks with a lateral stripe evident posteriorly but fading anteriorly and not reaching the head. Most of the habitats for the species, which are given above are now drought or are under sever human- and natural-dependent treats [2,7]. The recent efforts by the authors were not successful for observing of the species The aim of this study was (I) to add new mtDNA sequences of the Persian chub from the Kor and two new localities in Maharlu and Persis basins in Southern Iran to infer their phylogenetic relationships among the numerous species of the subfamily Leuciscinae available in genbank, (II) to add new distribution record, and discuss its zoogeographic distribution in southern Iran and (III) to provide information on conservation status of the Persian chub.

## MATERIALS AND METHODS

**Study region**: The Persian chub specimens were collected from its distribution range from Kor, Maharlu and Persis basins using electrofishing device. After anesthesia, the fin clips and muscle tissues were removed and were fixed in 96% alcohol for molecular analysis. Materials for this study are from the previous available published data [2], and from the extensive fieldworks by authors that provided the *geographic* *coordinate* *datasets* *for* *Persian chub* *distribution (mainly from ZM-CBSU)*. The distribution of Persian chub from entire drainage basins of Iran was mapped by using DIVA-GIS (7.5.0) software [13].

**DNA extraction, PCR amplification and phylogenetic analyses**: Total genomic DNA was extracted according to phenol/chloroform procedures [14]. The partial mitochondrial cytochrome b gene was amplified by polymerase chain reaction (PCR) using either the primers Glu-F (5'-AAC CAC CGT TGT ATT CAA CTA CAA-3') and ThrR (5'-CCT CCG ATC TTC GGA TTA CAA GAC CG-3' [15], or L14724 (5'-GTG ACT TGA AAA ACC ACC GTT G-3') and H15915 (5'- CAA CGA TCT CCG GTT TAG AAG AC-3', [16,17]). Amplification was performed in a thermal cycler programmed as following: initial denaturation 94 °C for 3 min, 35 cycles with denaturation at 94 °C for 50 s, annealing 56 °C for 45 s, extension 72 °C for 1 min per cycle and followed by a final extension phase at 72 °C for 5 min. New sequences were edited in Bioedit and are deposited in NCBI Genbank (www.ncbi.nlm.nih.gov). Accession numbers for the new materials and materials from NCBI are given in Table 1. The new sequences with the additional sequences of the subfamily Leuciscinae published in Genbank were aligned by using Muscle 3.6 [18], as incorporated in Geneious, under default settings in order to gain a representative data set for the assessment of the phylogenetic position of the Persian chub in southern Iran. Maximum likelihood reconstructions were performed using RAxML 7.2.5 [19] under the GTR+G+I model of nucleotide substitution, with CAT approximation of rate heterogeneity and fast bootstrap (2000 bootstrap replicates). Bayesian likelihood phylogenetic analyses of nucleotide sequences were run with the version of MrBayes 3.1.2 [20] under the most generalizing model (GTR+G+I) because overparametrization apparently does not negatively affect Bayesian analyses [21]. Analyses were terminated after the chains converged significantly, as indicated by the average standard deviation of split frequencies < 0.01. The evolutionary divergence over sequence pairs between groups of the studied species were estimated using the Kimura 2-parameter model [22].

**Table 1:** Accession numbers for the new materials and materials from NCBI

	**GenBank No.**	**Species**	**Subfamily**
1	DQ350254	*Alburnus alburnus*	Alburninae
2	HM173165	*Alburnus thessalicus*	Alburninae
3	HM560071-2	*Alburnus kotschyi*	Alburninae
4	HM560069-70	*Alburnus filippii *	Alburninae
5	HM560067-68	*Alburnus escherichii *	Alburninae
6	HM560065	*Alburnus belvica*	Alburninae
7	HM560063-64	*Alburnus arborella*	Alburninae
8	HQ167604	*Alburnus orontis*	Alburninae
9	HM560066	*Alburnus *sp.	Alburninae
10	AY509846-48	*Scardinius erythrophthalmus*	Leuciscinae
11	AY509833	*Scardinius scardafa*	Leuciscinae
12	AY509832	*Scardinius graecus*	Leuciscinae
13	AY509831	*Scardinius acarnanicus *	Leuciscinae
14	HM560174	*Scardinius hesperidicus*	Leuciscinae
15	HM560176	*Scardinius plotizza*	Leuciscinae
16	HM560170	*Scardinius acarnanicus*	Leuciscinae
17	AB162651	*Leuciscus waleckii*	Leuciscinae
18	HM560101	*Leuciscus leuciscus*	Leuciscinae
19	AY509823	*Leuciscus leuciscus*	Leuciscinae
20	HM560076-77	*Blicca bjoerkna*	Leuciscinae
21	AY026409	*Abramis ballerus*	Leuciscinae
22	AY026411	*Acanthobrama marmid*	Leuciscinae
23	AY026406	*Acanthobrama terraesanctae*	Leuciscinae
24	HM560056	*Acanthobrama lissneri*	Leuciscinae
25	AY026407	*Acanthalburnus microlepis*	Leuciscinae
26	KT321511-16	*Acanthobrama persidis*	Leuciscinae
27	HM560112-13	*Acanthobrama persidis*	Leuciscinae
28	AF090749	*Chondrostoma vardarense*	Leuciscinae
29	AF533760	*Chondrostoma nasus*	Leuciscinae
30	AF533764	*Chondrostoma holmwoodii*	Leuciscinae
31	DQ447735	*Chondrostoma prespense*	Leuciscinae
32	HM560084	*Chondrostoma oxyrhynchum*	Leuciscinae
33	AF533757	*Chondrostoma regium*	Leuciscinae
34	AY026400	*Chondrostoma regium*	Leuciscinae
35	AY494765	*Pseudophoxinus battalgilae*	Leuciscinae
36	HM560128	*Pseudophoxinus alii*	Leuciscinae
37	AY494752	*Pseudophoxinus anatolicus*	Leuciscinae
38	AY494755	*Pseudophoxinus maeandri*	Leuciscinae
39	AY494762	*Pseudophoxinus antalyae*	Leuciscinae
40	AY494763	*Pseudophoxinus crassus*	Leuciscinae
41	AY494768	*Pseudophoxinus kervillei*	Leuciscinae
42	AJ698710	*Squalius alburnoides*	Leuciscinae
43	AJ698717	*Squalius pyrenaicus*	Leuciscinae
44	AJ698711	*Squalius aradensis*	Leuciscinae
45	AJ852495	*Squalius aradensis*	Leuciscinae
46	AJ698456	*Squalius carolitertii *	Leuciscinae
47	AF090741	*Alburnoides ohridanus*	Leuciscinae
48	HM173170	*Alburnoides fasciatus*	Leuciscinae
49	HM173163	*Alburnoides prespensis*	Leuciscinae
50	HM173167	*Alburnoides bipunctatus*	Leuciscinae
51	AB236729	*Phoxinus steindachneri*	Leuciscinae
52	EU755036	*Phoxinus phoxinus*	Leuciscinae
53	AB236730	*Phoxinus oxycephalus*	Leuciscinae
54	HM560122	*Phoxinus bigerri*	Leuciscinae
55	HM560123	*Phoxinus lumaireul*	Leuciscinae
56	HM560111	*Petroleuciscus borysthenicus*	Leuciscinae
57	GU131228	*Petroleuciscus borysthenicus*	Leuciscinae
58	HM560114	*Petroleuciscus smyrnaeus*	Leuciscinae
59	HQ167619	*Petroleuciscus smyrnaeus*	Leuciscinae

**Species Distribution Modeling**: Species Distribution Modelling (SDM) was used to predict the climatically suitable habitats for *Acanthobrama persidis *in Iran. To construct the model, 19 climatic variables including temperature and precipitation layers obtained from the WordClim data set with 30-second spatial resolution [23] were combined to georeferenced occurrence locations for the species from Iran. Model was constructed using the maximum entropy algorithm implemented in MaxEnt 3.3.3 software.

At first, we run the models with all climatic variables to diagnose the more informative variables. Environmental information from 500 randomly generated geographic points across the study area was extracted in order to establish a set of uncorrelated climatic layers. The more biologically informative variables (based on the species ecological requirements, a pairwise Pearson correlation matrix, and variables with more permutation importance to construct the model recognized from initially run with all layers) were retained in this step including seven variables with R^2^ < 0.90 as following; Bio 2: mean diurnal temperature range, Bio 8: mean temperature of wettest quarter, Bio 9: mean temperature of driest quarter, Bio 12: annual precipitation, Bio 15: precipitation seasonality, Bio 16: precipitation of wettest quarter and Bio 18: precipitation of warmest quarter. Predictive model was evaluated by splitting localities into 75% training and 25% test data. All Maxent runs were adjusted with a convergence threshold of 1.0E-5 with 1000 iterations, application of a random seed, logistic probabilities and other settings were left at default levels. The models were tested using the AUC (area under the receiver operating curve) approach of Phillips et al. (2006) [24] and mean value ≥ 0.7 as evidence that the model had sufficient discriminatory ability [25]. Resulting ASCII files imported into DIVA-GIS (available at http://www.diva-gis.org/) to visualize the models. 

## Results

**Figure 3:**
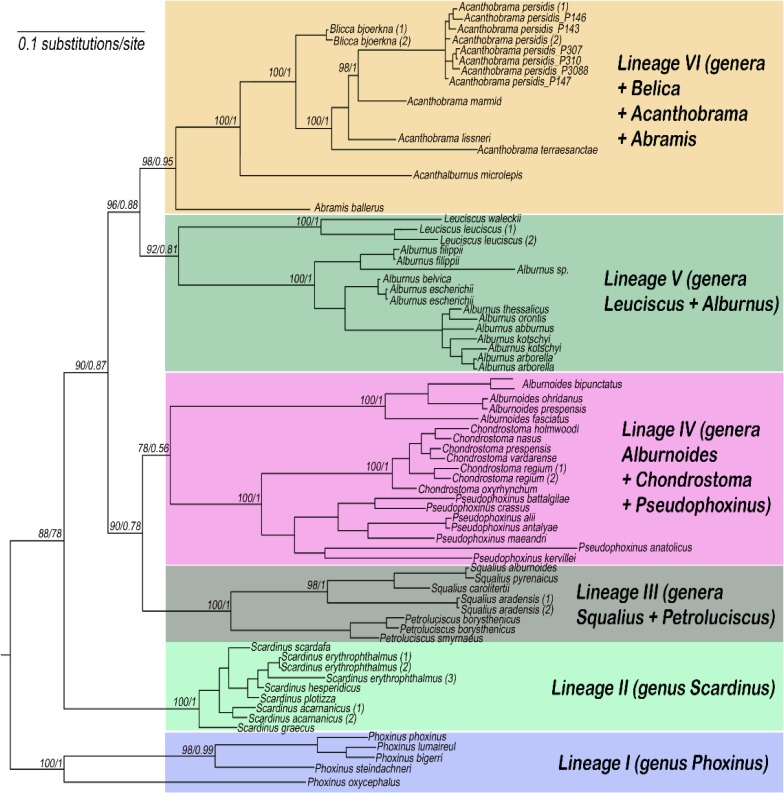
Maximum likelihood estimate (based on cytochrome b gene sequences) of phylogenetic relationships of the studied *Acanthobrama persidis* and related taxa. Numbers above branches represent maximum likelihood bootstrap values based on 2000 replicates followed by Bayesian posterior probabilities

**Mitochondrial phylogenetic relationships**: All mitochondrial analyses generated almost identical and well-supported topologies. However, since Bayesian analysis has been suggested to be the most efficient character-based method for accurately reconstructing a phylogeny, we focused our discussion on Bayesian tree. The results of the Maximum likelihood (ML) tree have also considered in our discussion. According to molecular mitochondrial cyt b data of our study, the studied taxa classified in six major lineages. All lineages were supported by high posterior probability and bootstrap values (Fig. 3).

**Lineage I.** linage 1 was formed by the monotypic genus of the *Phoxinus* (sub-family Phoxininae) which include several species and subspecies; *Phoxinus steindachneri*, *P. phoxinus*, *P. lumaireul*, *P. bigerri* and *P. oxycephalus jouyi*.

**Lineage II.** It comprised the species of the genus *Scardinus* including; *S. erythrophthalmus*, *S. scardafa*, *S. hesperdicus*, *S. plotizza*, *S. graecus*.

**Lineage III.** It includes five species of the genus *Squalius* (*S. alburnoides*, *S. pyrenaicus*, *S. aradensis*, S*. carolitertii* and *S. aradensis*) and three species of the genus *Petroluciscus* (*Petroleuciscus smyrnaeus*, *P. borysthenicus* and *P. smyrnaeus*).

**Lineage IV.** It is a big clade, which comprised species of the three genus *Alburnoides *(*A. fasciatus, A. ohridanus, A. strymonicus,* *Alburnoides prespensis*); *Chondrostoma *(*Chondrostoma regium, C. vardarense, C. oxyrhynchum,* *C. prespense, C. nasus *and* C. holmwoodii*); *Pesudophoxinus *(*Pseudophoxinus maeandri, P. anatolicus, P. alii *and* P. battalgilae*). In this clade species of the genus *Chondrostoma* form a sister group to the species of the genus *Pesudophoxinus*, and this two together are sister to species of the genus *Alburnoides*.

**Lineage V.** This linage included species of the two genera *Alburnus* (*Alburnus arborella*, *A. orontis*, *A. arborella*, *A. belvica*, *A. escherichii*, *A. escherichii*, *A. filippii*, *A. kotschyi*, *A. kotschyi*, *A. thessalicus*, and *A. alburnus*) and *Leuciscus* (*Leuciscus leuciscus*, *L. leuciscus* and *L. waleckii*), in which they form sister group. 

**Lineage VI.** It comprised of three genera *Abramis *(*Abramis brama *and* A. ballerus*); *Acanthobrama *(*Acanthobrama marmid*, *A. terraesanctae*,* A. lissneri*), and *Belica *(*Blicca bjoerkna *and* B. bjoerkna*). In addition to these genera, our new sequences – P147, P147, P307, P308, P3010 – from the drainages in southern Iran (Kor, Helleh and Hormuzgan), plus two sequences of *Petroleuciscus* *persidis* (Accession numbers HM560113 and HM560112) from GenBank are also categorized in this lineage. The new sequences from southern Iran and the two sequences of the *P.* *persidis* from GenBank form sister group to the genus *Acanthobrama*. 

**Distribution and new record for** ***Acanthobrama*** **in Southern Iran**: The type locality of *Pseudophoxinus persidis* is the "upper Shur River drainage at "Koorsiah" village (upper part of the Hormuzgan Basin), near Darab on Darab-Fasa road, 28°45.5'N, 54°24'E, Fars" [2]. The other locality, which Persian Chub has been recorded is from a village 32 km west of Kor River Bridge on road to Dariush Dam, 30°04.5'N, 52°36'E, Fars" [2]. Till date, only these localities are given for this species. However, in this study we added two another localities for this species in southern Iran including Maharlu Lake basin and Arjan Wetland in upper part of the Helleh River (Persis basin), which is located among the Zagros Mountains (Fig. 1).

**Figure 4:**
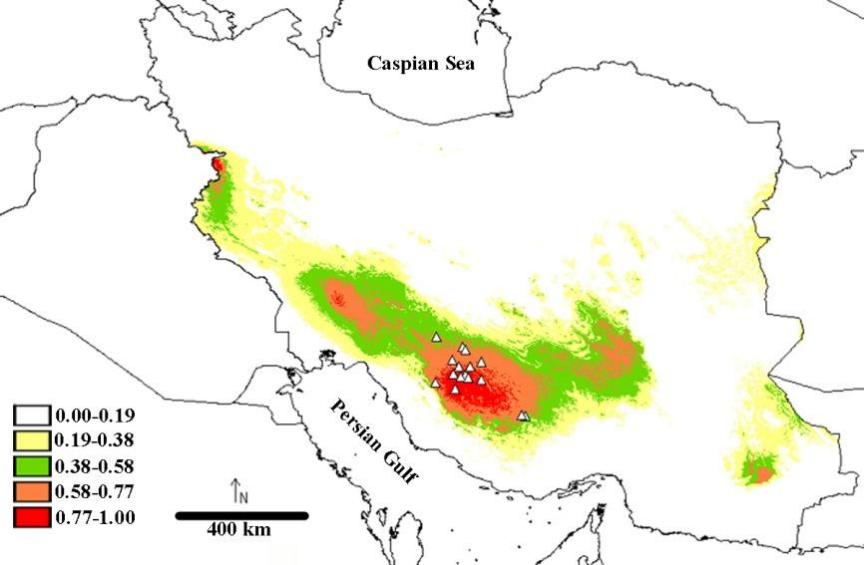
Potential distribution of *A. persidis *in Iran using MaxEnt. Sampling point localities are indicated with hollow triangular. Red colors indicate higher habitat suitability for *A. persidis*

The predicted geographic distributions for the species based on current bioclimatic variables are visualized in Figure 4. The AUC value (AUC _Test_= 0.97) supported the discriminative power of the model. The model showed a large area of suitable climate for *A. persidis *across south and west of Iran. Climatic conditions are highly suitable for the species in Kor river basin and it was predictable (Figure 4). Some other parts in the Persis and Tigris basins might also been suitable habitats for this cyprinid species showing possible dispersal route of *Acanthobrama* from Tigris to the Persis, Kor and Kol basins.

**Habitat**: This species was collected in large rivers, streams, springs, irrigation ditches and qanats [7], but all these have in common a stream-like environment for much of the year. They are relatively shallow (20 cm to 2 m), variable width (0.5-75 m), medium to slow current, some submergent and emergent aquatic vegetation and a bottom varying from pebbles and gravel to mud. Water temperatures varied from 15 to 23°C from October to January and presumably would be over 30°C in the summer. Conductivity ranged from 0.3 to 1.0 mS. Altitude ranged from 980 to 1940 m. The lower reaches of some of the Capture Rivers are salty and may not support this species [7].

**Figure 5:**
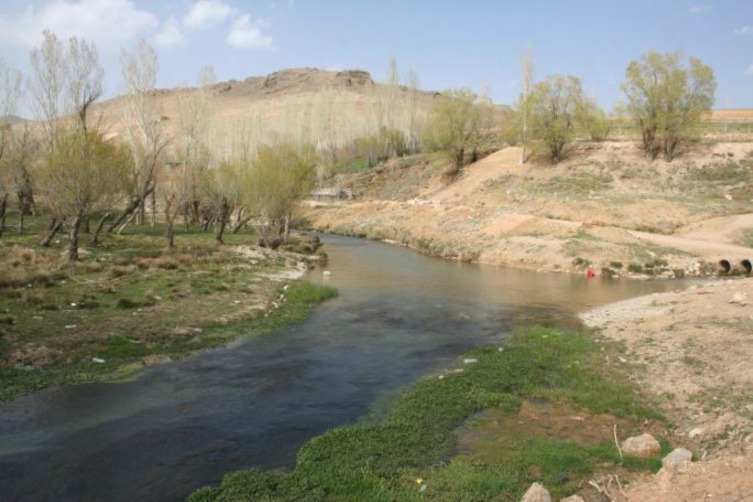
Natural habitat of *Acanthobrama persidis* in upper reaches of Kor River basin

In the uppermost reaches of the Kor River Basin, in Khosroshirin stream and its connected springs including Paselari and Pahn springs (30°53′ 57.4˝ N. 51°59′52.5˝ E.), Persian chub founds in altitude of 2329 (Fig. 5). The substrate of the Paselari spring is generally muddy with small gravels, and the water surface is covered by dense filamentous green algae. The surrounding area is vegetated with reed (*Phragmatis* sp.), rush (*Juncus* sp.), shrubs and trees. Water current is high in Khosroshirin stream and low in Paselari and Pahn springs. The mean value of chemo-physical analysis of three stations from Paselari spring includes NO-3 1.95 mg l-1, NO-2 0.42 mg l-1, PO_4_^-3^ 0.59 mg l-1, NH‏3 0.12 mg l-1, DO 8.92 mg-1, DO% 122.6, TDS 191.7 mg-1, conductivity 395 ls cm-1, salinity 0.180/00, pH 7.9 and water temperature 17.05°C. 

**Co-exist species**:* Alburnus mossulensis*, *Capoeta aculeata*, *Capoeta saadii, Carrasius auratus, Cyprinus carpio, Hypophthalmichthys molitriox, H. nobilis*,* Pseudorasbora parva, *(Cyprinidae); *Cobitis linea* (Cobitidae); *Oxynoemacheilus* *persa*, *O. tongiorgii*, *Paracobitis persa*  (Nemacheilidae);* Aphanius farsicus*, *A.* *shirini*,* A. sophiae *(Cyprinodontidae);* Gambusia* *holbrooki *(Poecilidae);* Chelon abu *(former considered as* Liza abu*) (Mugilidae);* Onchorhynchus mykiss *(Salmonidae), have been found to be co-exist with *A. persidis *in streams and springs in its entire distribution range. 

## Discussion

**Taxonomy of the** ***Acanthobrama*** ***persidis* in southern Iran resulted from mtDNA phylogeny: **Iranian *Petroleuciscus persidis* was initially described as a species of *Pseudophoxinus *[2]. Later it was transferred to the genus *Leuciscus* [26] suspected to be related to *P. smyrnaeus *(former considered as *L. smyrnaeus*) [27], then included in *Petroleuciscus* [28]. It was transferred to the genus *Squalius *[29]. These relationships are inconsistent with our molecular data and Perea et al. (2010) [12], which strongly support close affinities of *P. persidis* with the genus *Acanthobrama*, also pointing on the difficulties of morphological characterization of miniaturized cyprinids [30, 31]. As results and based on our new molecular phylogenies, the examined sequences of the Persian Chub from drainages of southern Iran fall in the clade of the genus *Acanthobrama*, while the sequences from two other genera (*Pseudophoxinus* and *Petroleuciscus*) are in distinct clade and phylogenetically far from the genus *Acanthobrama*. Also, two sequences of the Persian chub from southern Iran (which is already deposited in GenBank in the name of Petroleuciscus* persidis *with accession numbers HM560113 and HM560112) are clustered in the clade of the genus *Acanthobrama.* Therefore, we concluded that the populations of Persian chub in drainages of southern Iran (i.e., Kor, Hormuzgan and Helleh) belong to the genus *Acanthobrama *and species* Acanthobrama persidis. *

 Furthermore, our data support Perea et al. (2010) [12], in the synonymization of the genus *Acanthalburnus* with *Acanthobrama*, both genera just distinguished by the two vs. one rows of pharyngeal teeth [32].

*The taxonomic status of two other nominal species in Iran, namely *Petroleuciscus ulanus *(Günther, 1899) [*10*] and *P*. esfahani *Coad &* Bogutskaya, 2010 [*9*] should be re-assessed using molecular data. The taxonomic status of *Petroleuciscus gaderanus *(Günther, 1899) [*10*] in Lake Orumiyeh basin which has been tentatively considered as synonym of *P. ulanus *[*7*] also should be re-assessed. A fourth geographically close species is *P. kurui *(Bogutskaya, 1995) [*33*] from the upper Tigris River basin in southeastern Turkey should be analyzed. There are three more species in the genus i.e., *P. squaliusculus *(Kessler, 1872) in Syr Darya drainage, Kazakhstan, Kyrgyzstan and Tadjikistan; *P. smyrnaeus *(Boulenger, 1896) in western Turkey; and *P. borysthenicus *(Kessler, 1859).* Based on our phylogenetic analysis and Perea et al. (2010) [12] who used mitochondrial and nuclear DNA sequences, both P. smyrnaeus *and *P. borysthenicus* (type species of the genus, a circum-Pontic species) remain in the genus *Petroleuciscus.

In their study, Perea et al. (2010) [12] include *P. persidis* with *Abramis, Acanthobrama, Acanthalburnus, Ballerus, Blicca, Mirogrex* and *Vimba.* Durand et al. (2002) [34], Perea et al. (2010) [12] and Geiger et al. (2014) [35], supported the hypothesis that *Mirogrex* is indeed a distinct genus. The molecular analysis presented here and those published by Durand et al. (2002) [34] and Perea et al. (2010) [12] revealed that *Acanthalburnus microlepis* from the southern Caspian Sea basin to be nested within *Acanthobrama *and close to *A. marmid*, the type species of *Acanthobrama*. The two genera are distinguished by the number of rows of pharyngeal teeth (two in *Acanthalburnus* vs. one in *Acanthobrama*), a character known to vary between species within a genus in Cyprinidae. For example, in teleost, most species have two rows, but four species are known to have a single row [36, 37]. Moreover, *Acanthalburnus microlepis *and the closely related *Acanthalburnus urmianus* from northern Iran are therefore included in *Acanthobrama*. As results and based on total evidence approach, eleven species are recognised as valid in the genus *Acanthobrama* (*A. centisquama*, *A.* *hadiyahensis*, *A. lissneri*, *A. marmid*, *A. microlepis*, *A. orontis*, *A. persidis*, *A. telavivensis*, *A. thisbae,* *A. tricolor*, *A. urmianus*) [38]. *Acanthalburnus *is treated as a synonym of *Acanthobrama *[38].

**Biogeographical Considerations:** *Acanthobrama* is a northern genus of the subfamily Leuciscinae and its colonization in the Persis basin should have come from Mesopotamia - but when these species evolved, Mesopotamia was very different and the fishes must have gone through river connections through the Zagros mountains.‏

To understand the distribution pattern and biogeography of the *A. persidis* and related species we need to have information about I) Iranian basins which are inhabited by *A. persidis* and its relatives, II) their connections in the past and III) discussing origin and initial colonization of Leuciscinae based on the available literatures.

I) **Iranian basins which are inhabited by *A. persidis* and its relatives:** Based on the collected distribution data, *A. persidis* populations in Iran are found in the following basins:

**Hormuzgan Basin:** This basin (Fig. 1) in the Zagros Coastal Plain or Central Zagros is delimited to the west by the North Zagros and to the east by the Makran Basin [39]. The Hormuzgan Basin probably is separated from the other units of the Zagros Mountains since the late Pliocene and Pleistocene [39]. The geologic events within the Hormuzgan Basin are responsible for the presence of many unusual and isolated habitats (e.g. hot springs). This habitat fragmentation probably increased the species diversity among fishes (see [1]). The Hormuz (or Hormozgan) basin comprises a number of intermittent streams and rivers which drain to the Straits of Hormuz. The principal river is the Kul with its tributary the Shur (= salt) River. 

**Maharlu Basin:** The Maharlu Basin (Fig. 1) is a tectonically constrained endorheic basin within the Simple Folded Belt of the Zagros Mountains [40-41]. It lies close to the Sabz–Pushan Fault and forms part of the Kazerun Fault System, which is one of the most active fault systems in southern Iran and includes the Kazerun, Kareh–Bas, Sabz–Pushan and Sarvestan faults [Fig. 1]. The formation of the Maharlu Basin is probably related to the tectonic activity that characterized the geological history of the Zagros Mountains during the Pliocene (5⋅0–1⋅8M b.p.) and Pleistocene (1⋅8M b.p.–10 000 b.p.) [39, 42]. These events led to rapid isolation of multiple areas, created new barriers to migration and altered hydrological networks [40]. The young geological age of the Maharlu Basin is additionally supported by its elongated shape, which is typical of basins located in tectonically active mountain ranges [42-43] (Fig. 1).

**Kor Basin:** Major rivers are the Kor (= the classical Araxes) and its tributary the Pulvar (or Sivand) (= the classical Medus) which rise in the Zagros Mountains to the north and north-west and drain to the north-west corner of Lake Tashk. The Kor River Basin principally belongs to the High Zagros; altitudes are between 1557 and 3978 m above sea level [44]. Its geological history is associated with the geological evolution of the Zagros Mountains, which characterize the collision zone between the Arabian and Eurasian plates [39,45]. The collision probably started between 35 and 20 Ma ago [46]. However, a prominent phase of tectonic deformation occurred in the Late Miocene and Early Pliocene (ca. 10–5 Ma ago) and led to the rise of the Iranian plateau and new configurations of mountain ranges and drainage patterns [40, 47]. As the Kor River Basin is located just within the collision zone (Fig. 1), its initial configuration is probably linked to this Late Miocene–Early Pliocene period of geological activity. Notably, the initial Kor River Basin was exorheic and drained to the Persian Gulf [44, 48]. The present-day Kor River Basin is endorheic and considerably younger; it is probably formed in the Late Pleistocene (20 000–10 000 years ago, Nadji, 1997) or Holocene (6000–2000 years ago, Loffler, 1959). 

**Persis Basin:** This basin (Fig. 1) comprises rivers which drain the southern Zagros Mountains to the head of the Persian Gulf, but which are not now tributaries of the Tigris River nor are they the salt streams of Hormuz. At its northern edge, the Zohreh River flows across the Khuzestan plains and is close to Tigris River tributaries. Other major rivers are the Helleh, which debouches into the Persian Gulf north of Bushehr (28°59'N, 50°50'E) and the Mond or Qarah Aqaj (= the classical Sitakos), which, with its tributaries, drains much of Fars Province to the Persian Gulf in south of Bushehr. 

II) **River drainage connections in the past:** Headwater capture is common in the Zagros Mountains [49] and in Anatolia and pluvial conditions in the past would have facilitated fish dispersal [50]. Headwaters of a number of Tigris-Euphrates rivers interdigitate with the upper reaches of Black-Caspian Seas basin, e.g. the Aras River of the Caspian Sea and the Kizilirmak of the Black Sea with the Euphrates near Erzurum and Silvas respectively; the Qezel Owzan (a tributary of Safidrud) of the Caspian Sea with Tigris tributaries [50].

Por and Dimentman (1989) [51] regard the Tigris-Euphrates or Mesopotamian basin as a cradle for inland aquatic faunas. A proto-Euphrates collected water from the Levant and had contacts with the Black and Caspian Sea drainages before the Pliocene orogeny. Berg (1940) [52] points out that the upper reaches of the Tigris-Euphrates basin today lie on a plateau close to the upper reaches of the Caspian Sea basin. This headwater river capture may explain close relationship of *Acanthobrama microlepis *(Caspian Sea basin) and the closely related *Acanthobrama urmianus* (Urmia Basin) from northern Iran as seen in Figure 3. The basin acted as an area where African and Asian species could meet or transit such as the Iranian cichlid, *Iranocichla hormuzensis*. These connections were interrupted in the early Pliocene by orogeny, rifting and desert formation. Banarescu (1977) [53] and Por and Dimentman (1989) [51] regard the area to be a zoogeographic crossroads with elements from the Palaearctic such as the cyprinid genera *Leuciscus* (= *Squalius*) and *Chondrostoma*, Mediterranean genera such as the cyprinid *Acanthobrama* (although Krupp (1987) [26] refers to this genus as Palaearctic, of Mesopotamian origin), and Oriental genera such as the cyprinid *Garra* and the spiny eel *Mastacembelus*. Al-Rudainy (2008) [54] and Coad (2010) [55] are recent accounts of the fishes in Iraq.

**Figure 6:**
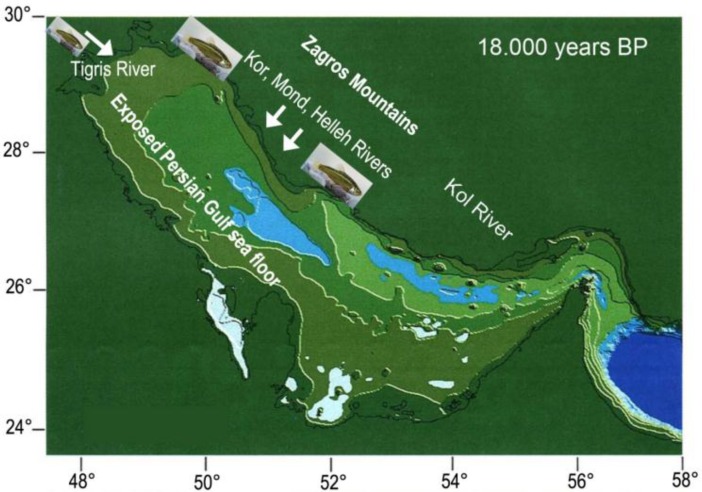
Exposed sea floor of the Persian Gulf Sea during the Last Glacial Maximum of the Late Pleistocene at 18.000 years BP, with possible freshwater areas in depressions (marked in blue); elevations above sea level are indicated with different greenish colours at 20, 50, 100 and 150 m (modified from Lambeck, 1996). Arrows indicate river runoff from the Zagros Mountains

Moreover, Tigris-Euphrates has been connected to the Persis and Kor basins. In the Pleistocene, Paleo-Kor River drained from the High Zagros into the Persian Gulf [56] connecting Kor basin to the Persis basin. The present-day biogeography of these freshwater species indicates instead that the Persis, Tigris and Kor Basin systems must have been connected to each other in the recent past (Fig. 6). A plausible scenario suggests that the three drainage systems formed a single network during the Last Glacial Maximum of the Late Pleistocene (21,000-18,000 y.b.p.). At that time, the floor of the Persian Gulf was exposed due to the global fall in sea level, and it is therefore not unlikely that the Tigris and Euphrates (Tigris Basin), the Mond, Helleh (Persis basin), Kol (Hormuz basin) and also the Kor River (ancient exorheic Kor Basin) extended onto what is now the floor of the Persian Gulf, where they came together (see [48, 56-59] (Fig. 6). This postulated interconnection, which may have lasted until the Holocene sea-level rise at around 11,000 y.b.p., offers a persuasive explanation for the similarities observed between the fish faunas in drainage systems that are now wholly isolated from each other (see [56]). This scenario may explain the close relationship of *Acanthobrama* persidis found in Kor, Persis and Kol basins to the *A. marmid* found in Tigris basin. Moreover, the headwaters of Kol and Mond drainages are very close.

III) **Origin and initial colonization of Leuciscinae: **Perea et al. (2010) [12] proposed an initial colonization of Europe from Southwestern Asia via the Balkanian/Anatolian/Iranian landmass at the beginning of the Early Oligocene, which precisely matches the initial splitting of the most basal leuciscins. According to them, later vicariant events as the Paratethys isolation and later reconnection with Tethys during the Oligocene and the Alpine Orogeny, Dinarid lake systems and Gomphoterium land bridges during Miocene promoted Leuciscinae diversification. Our data also corroborate the colonization of North Africa before the Messinian salinity crisis. In Upper Miocene the opening of Aegean Sea was an important vicariant event for Anatolian and Greek leuciscins. Messinian appears as a stage of gradually Leuciscinae diversification more than a critical point of speciation.

The origin and dispersion of Leuciscinae lineages has also been discussed [34]. Based on their data, Durand et al. (2002) [34] concluded that all Leuciscinae lineages sampled in the Middle East also occur in the Euro-Mediterranean region. Furthermore, Tsigenopoulos (1999) [60] has shown that Middle Eastern *Luciobarbus *species have strong affinities with Euro-Mediterranean species. All these results are in agreement with authors such as Berg (1949) [40] and Coad (1996) [50] who considered that the Middle Eastern ichthyofauna is descended from the Euro-Mediterranean (Palaearctic) one. Given the sequence divergence between Middle Eastern and Mediterranean species of both Leuciscinae and *Luciobarbus*, and assuming an evolutionary rate of 1% per million years [61], these cyprinid species reached the Middle East at the end of the Late Miocene era. This is in accordance with the assumption of a freshwater fish dispersion during the “lago mare” phase of the salinity crisis in the Mediterranean Sea (5.5 My) [62]. According to Bianco (1990) [62] this important geological event allowed freshwater fish to disperse quickly through the oligohaline or freshwater Paratethys Sea through the Mediterranean Sea and lead to the present endemic ichthyofauna. This scenario was previously suggested for the interpretation of the multifurcation and/or divergence of Mediterranean species that belong to the *Leuciscus *subgenera *Squalius*, *Chondrostoma *and *Luciobarbus *([15, 60]). It has been [34] clearly indicated that the messinian dispersion was probably the most massive colonization event for the Middle Eastern. Based on the above literatures data it may conclude that *Acanthobrama* species found in Iran originated from Euro-Mediterranean region and then dispersed to the Tigris and then to the Persis and Kor basins. However, using different nuclear and mitochondrial markers are suggested to approve this hypothesis.

**Conservation aspects** **: Threats:** There is no recent record of this fish from its type locality in Kol river drainage (Hormuz basin). Its natural habitat in upstream of Helleh River (Persis basin) is being dried out. Its habitats in Maharlu basin is dried out or is under severe pressure. It seems that *A. persidis* populations are now restricted to small streams in upper reaches of Kor River basin. Water diversions severely reduce water level, especially pumping water from the aquifer which feeds the systems; drainage rehabilitation; water pollution from domestic and agricultural sources; severe drought in recent years; an intensive aquaculture industry which added a high nutrient load and several exotic fish species. The main threat is carnivorous exotic *O. mykiss* (rainbow trout), which escape from the fish farms in the upper reaches of Kor basin and enter to the stream.

**Conservation actions:** Some form of legal protection should be instituted; habitat monitoring, research on the biology and ecology of this endemic fish should be carried out and the maps of conservation hot spots should be drawn based on a combination of characters. Education of local people, especially students through the media and workshops should be initiated.

## Notes

**Acknowledgments: **We are thankful to Shiraz University for financial supports.
